# Early manifestation of alteration in cardiac function in dystrophin deficient mdx mouse using 3D CMR tagging

**DOI:** 10.1186/1532-429X-11-40

**Published:** 2009-10-22

**Authors:** Wei Li, Wei Liu, Jia Zhong, Xin Yu

**Affiliations:** 1 Department of Biomedical Engineering, Case Western Reserve University, Cleveland, Ohio, USA; 2 Department of Radiology, Case Western Reserve University, Cleveland, Ohio, USA; 3 Department of Physiology and Biophysics, Case Western Reserve University, Cleveland, Ohio, USA; 4 Case Center for Imaging Research, Case Western Reserve University, Cleveland, Ohio, USA; 5 Department of Biomedical Engineering, Washington University, St Louis, Missouri, USA

## Abstract

**Background:**

Duchenne muscular dystrophy (DMD) is caused by the absence of the cytoskeletal protein, dystrophin. In DMD patients, dilated cardiomyopathy leading to heart failure may occur during adolescence. However, early cardiac dysfunction is frequently undetected due to physical inactivity and generalized debilitation. The objective of this study is to determine the time course of cardiac functional alterations in mdx mouse, a mouse model of DMD, by evaluating regional ventricular function with CMR tagging.

**Methods:**

In vivo myocardial function was evaluated by 3D CMR tagging in mdx mice at early (2 months), middle (7 months) and late (10 months) stages of disease development. Global cardiac function, regional myocardial wall strains, and ventricular torsion were quantified. Myocardial lesions were assessed with Masson's trichrome staining.

**Results:**

Global contractile indexes were similar between mdx and C57BL/6 mice in each age group. Histology analysis showed that young mdx mice were free of myocardial lesions. Interstitial fibrosis was present in 7 month mdx mice, with further development into patches or transmural lesions at 10 months of age. As a result, 10 month mdx mice showed significantly reduced regional strain and torsion. However, young mdx mice showed an unexpected increase in regional strain and torsion, while 7 month mdx mice displayed similar regional ventricular function as the controls.

**Conclusion:**

Despite normal global ventricular function, CMR tagging detected a biphasic change in myocardial wall strain and torsion, with an initial increase at young age followed by progressive decrease at older ages. These results suggest that CMR tagging can provide more sensitive measures of functional alterations than global functional indexes in dystrophin-related cardiomyopathies.

## Background

Duchenne muscular dystrophy (DMD) is an X-linked severe progressive muscle wasting disease, which affects approximately 1 in 3500 male birth [1,2]. DMD is caused by the deficiency of a cytoskeletal protein, dystrophin, which is a component of the transmembrane dystrophin-glycoprotein complex (DGC). DGC plays an important role in maintaining the structural integrity of the cells by linking intracellular actin filaments to basal lamina [3,4]. The disruption of DGC structure due to dystrophin deficiency leads to dilated cardiomyopathy (DCM) that may occur during adolescence [5]. At an early stage, DMD patients usually do not present clinical cardiac symptoms because of their physical inactivity and generalized debilitation. However, a recent cardiovascular magnetic resonance (CMR) tagging study suggests that abnormal myocardial strain may develop long before the manifestation of global functional deterioration [6].

The *mdx *mouse carries a nonsense mutation in its dystrophin gene that eliminates the expression of the dystrophin protein. It thus has been a popular animal model for studying the pathophysiology of DMD [7,8]. The *mdx *mice exhibit many of the same histological features seen in DMD, including the degeneration and necrosis of myofibers with inflammatory infiltrates, followed by subsequent regeneration. However, disease progression in *mdx *mice is milder as compared to DMD patients. Only in relatively older *mdx *mice are the progressive degenerative changes observed [9]. Similarly, *mdx *mice manifest histological evidence of a cardiomyopathy, but no overt cardiac dysfunction has been found in the young mdx mice. Reduced global cardiac function [10] and dilated right ventricles [11] were only documented in relatively older mdx mice. In the current study, we sought to delineate alterations in cardiac function and structure during the course of DCM progression in mdx mice. Longitudinal evaluation of 3D regional myocardial wall motion, a potentially more sensitive measure of ventricular contractile behaviour, was performed with CMR tagging.

## Methods

### CMR

In vivo CMR was performed at the Biological MR Laboratory of Washington University Medical Center. Mdx mice and their age-matched C57BL/6 wildtype controls of 2, 7 and 10 months of age underwent CMR on a 4.7 T Varian INOVA system (Varian Associates, Palo Alto, CA) equipped with a gradient insert (60 G/cm, 10 cm inner diameter) as described previously [12]. A 2.5-cm surface coil was used for the imaging of mice. Briefly, mice were anesthetized with 0.7~1% isoflurane by a nose cone and placed into the coil in prone position. Electrodes were attached to front paws and the right leg for ECG gating and monitoring of vital signs. The animals were kept warm by blowing hot air into the magnet using a blow dryer. The heat flow and anaesthesia level were manually adjusted to keep the heart rate similar to that under conscious conditions. Animals were sacrificed at the end of imaging protocol for histological analysis. A total of 42 mice were scanned. The animal protocol was approved by the Animal Studies Committee of the Washington University Medical Center.

Tagged images of 5 short-axis (SA) slices were acquired from base to apex. The tagging sequence used a SPAMM-1331 sequence applied immediately after the ECG trigger, followed by gradient-echo cine sequence. Repetition time (TR) was adjusted according to the R-R interval of the heart such that a total of 15 frames were acquired during one cardiac cycle. Other imaging parameters were: echo-time (TE), 3 msec; data matrix, 256 × 128; field of view (FOV), 4 cm × 4 cm; slice thickness, 1 mm. Tag resolution was 0.6 mm with tagline thickness ranging from 0.2 to 0.3 mm. The acquired *k*-space data were zero-filled to yield a matrix size of 512 × 512, corresponding to an in-plane resolution of 78 μm × 78 μm. Two sets of tagged SA images were acquired with tags in perpendicular directions for each SA plane, yielding a grid tagging pattern when the two data sets were combined. Tagged long-axis (LA) images were acquired from four radially distributed LA views spaced every 45°, with the tags perpendicular to the LV long-axis. Imaging parameters were the same as those used for SA images.

Regular cine images that provided better contrast between the myocardium and the blood were acquired for morphological analysis. These images were acquired with the same imaging parameters as the tagged images with a 128 × 128 data matrix. Both the cine and the tagged images were zero-filled into a 512 × 512 data matrix so that myocardial contours traced from cine images were directly used in the analysis of tagged images with minimal adjustment. Total image acquisition time, including animal setup, was ~2 hours.

### Image Analysis

**Figure 1:**
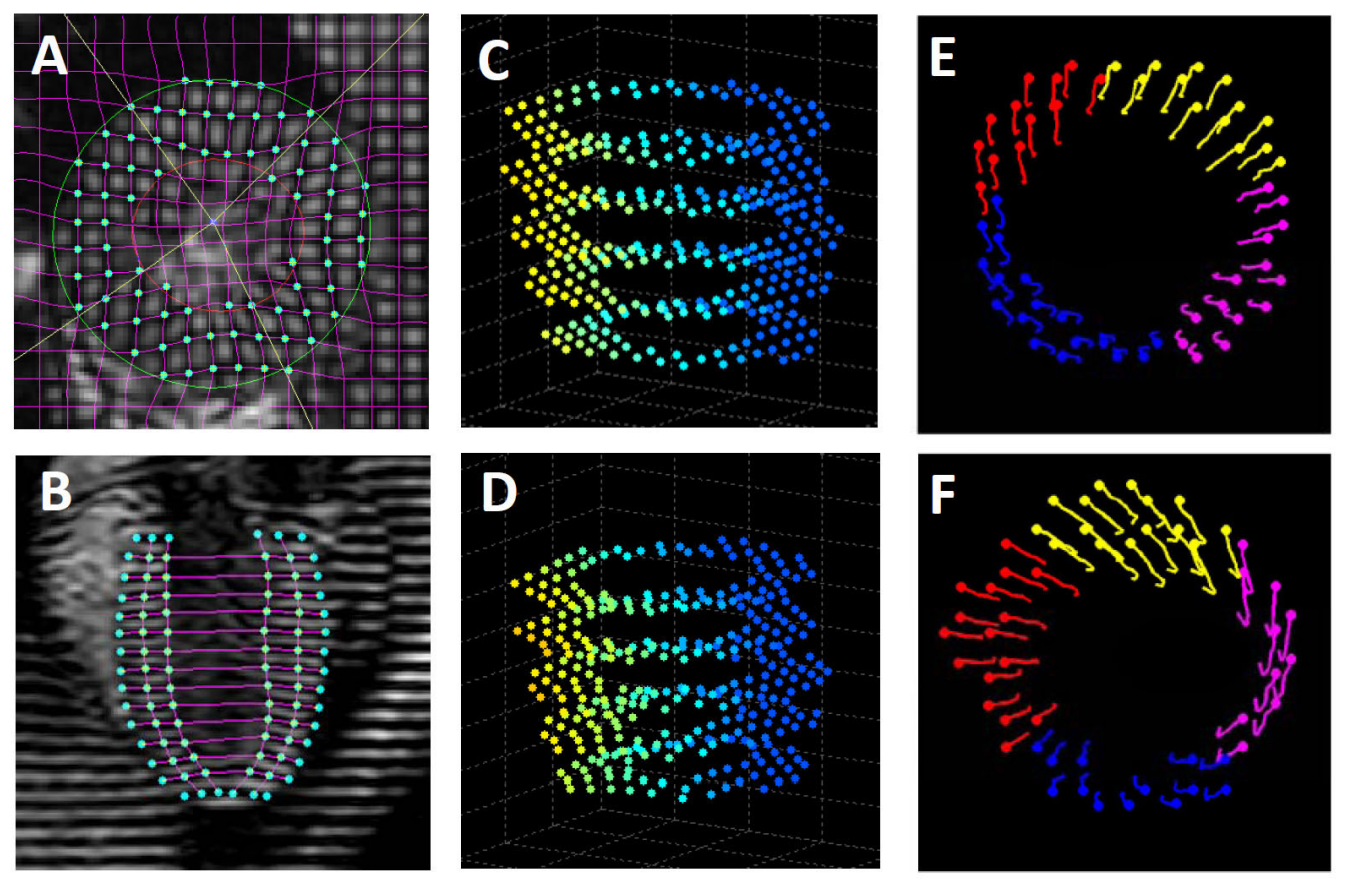
**3D MRI tagging image analysis**. A&B. Tracking of taglines in tagged short- and long-axis images, respectively; C&D. Reconstructed 3D tag points at end-diastole and end-systole, respectively; E&F. Trajectories of regional myocardial wall motion from end-diastole (dots) to end-systole (tails) at base and apex, respectively.

CMR data were analyzed with an in-house developed MATLAB-based software (CVMRI) described previously [12-15]. Epicardial and endocardial borders for SA images were traced interactively for the calculation of stroke volume, ejection fraction, and wall thickness. The left ventricular wall was further divided into four segments: septum, posterior, lateral, and anterior. Taglines for both SA and LA images were tracked semi-automatically with a HARP-based approach (Fig. 1A, B). Reconstruction of the 3D tag points was performed by first registering the SA and LA images in a 3D Cartesian coordinate system. Displacement of the intersecting tag points were tracked using an iterative point-tracking technique described previously [12]. The left ventricle was then divided into tetrahedrons using four non-coplanar adjacent tag points as the vertices. The 3D Lagrangian strain tensor was calculated from the deformation of these tetrahedrons, which was further diagonalized to yield principal strains E_1_, E_2_, and E_3_, with E_1 _and E_3 _being the maximal and minimal strains, respectively. The strain tensor was also transformed into a local myocardial coordinate system defined by the radial, circumferential, and longitudinal directions, yielding three normal strains in the circumferential (E_cc_), radial (E_rr_) and longitudinal (E_ll_) directions, respectively. Myocardial twist was computed as the rotation angle around the center of LV cavity (Fig. 1E &1F). Net twist angle was defined as the difference between the ventricular twist at apical and basal slices. Torsion was calculated as the net twist angle normalized by the slice separation. Typical processing time for a complete dataset was ~3 hours.

### Histology

**Figure 2:**
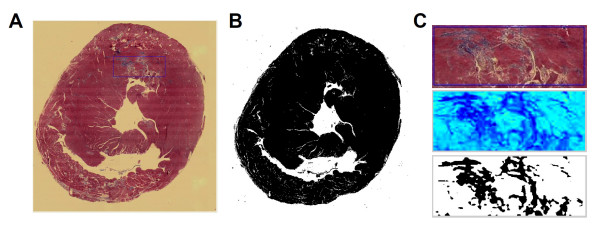
**Quantification of myocardial fibrosis**. A. Original histological image; B. Binary image of total tissue; C. Quantification of fibrotic tissue within a selected region of interest. Upper panel: original image; mid panel: pseudo-colored image of the difference between the red and blue channels; lower panel: binary image of fibrotic tissue.

Upon the completion of CMR, the mouse was sacrificed with an overdose of KCl injection. The heart was excised and fixed in neutral-buffered 10% formalin for histological analysis. The fixed heart was sliced in 1-mm increment from base to apex along the LV long-axis. Each slice was embedded in paraffin and sectioned at 4 μm. Tissue sections were stained with Masson's trichrome for the identification of fibrotic lesions. The extent of myocardial fibrosis was quantified from histological images using a thresholding method developed in MATLAB (Fig. 2). Pixels representing fibrotic tissue were extracted from the difference image of the red and blue channels of the original image, which provided the best contrast between normal and fibrotic tissue (Fig. 2C). Total myocardial tissue (normal and fibrotic) was extracted from the grayscale image (Fig. 2B). The percentage of tissue fibrosis was calculated as the ratio of the pixel numbers representing fibrotic and total myocardial tissue, respectively.

### Statistics

Data are presented as mean ± standard deviation. Unpaired Student t-test was used for comparisons between mdx mice and their age-matched controls. Mean values of myocardial strain in septal, anterior, lateral, and posterior segments, or at basal, midventricular, and apical levels, were compared separately by one-way analysis of variance (ANOVA). If there were statistical differences, multiple pairwise comparisons were performed using Tukey's test with a confidence interval of 95%. P values less than 0.05 were considered statistically significant.

## Results

### Animal Characteristics and Global Contractile Indexes

The animal characteristic data and global ventricular function measured from CMR are summarized in Table 1. Heart rate was similar to that reported in the literature at normal body temperature [16-18]. Heart rate, LV end-diastolic volume (EDV), LV end-systolic volume (ESV), stroke volume, ejection fraction and cardiac output were all similar between mdx mice and their age-matched controls in all three age groups. 10-month mdx mice showed an increase in both heart weight and body weight, but similar heart weight to body weight ratio. In addition, there was also a slight increase in wall thickness in 10-month mdx mice. However, the wall thickness to LV diameter ratio was similar.

**Table 1:** Animal Characteristics and Morphological Data.

	**2 month**		**7 month**		**10 month**	
	
	**control (n = 8)**	**mdx (n = 8)**	**control (n = 5)**	**mdx (n = 6)**	**control (n = 7)**	**mdx (n = 8)**
Age, weeks	8.5 ± 1.3	9.2 ± 1.5	30.6 ± 1.8	33.7 ± 0.7	45.2 ± 2.8	43.5 ± 1.2
Body weight, g	26.9 ± 1.8	23.5 ± 4.1	32.2 ± 5.3	34.2 ± 1.0	30.0 ± 2.9	34.5 ± 2.0^‡^
Heart weight, mg	141 ± 20	127 ± 13	137 ± 6	158 ± 29	148 ± 12	170 ± 12^‡^
Heart weight/body weight, %	0.52 ± 0.05	0.55 ± 0.13	0.43 ± 0.06	0.46 ± 0.09	0.49 ± 0.03	0.49 ± 0.03
Heart rate, BPM	466 ± 42	441 ± 40	478 ± 38	481 ± 54	483 ± 29	500 ± 22
LV diameter, mm	5.37 ± 0.18	5.07 ± 0.35	5.43 ± 0.19	5.71+ 0.12	5.73 ± 0.25	5.75 ± 0.08
Wall thickness, mm	0.97 ± 0.16	0.81 ± 0.05	1.02 ± 0.08	1.04 ± 0.09	0.94 ± 0.04	1.04 ± 0.09*
Wall thickness/diameter	0.18 ± 0.03	0.16 ± 0.01	0.20 ± 0.03	0.18 ± 0.02	0.17 ± 0.01	0.19 ± 0.02
LVEDV, μl	42.8 ± 6.7	40.6 ± 7.4	42.1 ± 7.4	48.2 ± 6.7	51.0 ± 7.2	47.8 ± 4.2
LVESV, μl	15.3 ± 5.0	13.7 ± 3.5	18.6 ± 8.4	20.9 ± 5.3	24.0 ± 6.7	24.3 ± 2.9
Stroke volume, μl	27.5 ± 3.1	26.9 ± 4.3	25.9 ± 0.5	27.3 ± 2.3	27.0 ± 3.2	23.5 ± 3.1
Ejection fraction, %	65 ± 7	66 ± 4	63 ± 10	57 ± 5	54 ± 8	49 ± 5
Cardiac output, ml/min	12.9 ± 1.5	11.8 ± 1.6	12.7 ± 0.9	13.4 ± 1.3	13.0 ± 2.2	11.7 ± 1.4

*P < 0.05, ^‡^P < 0.01 between mdx mice and age matched controls.

### 2-month mdx mice

**Figure 3:**
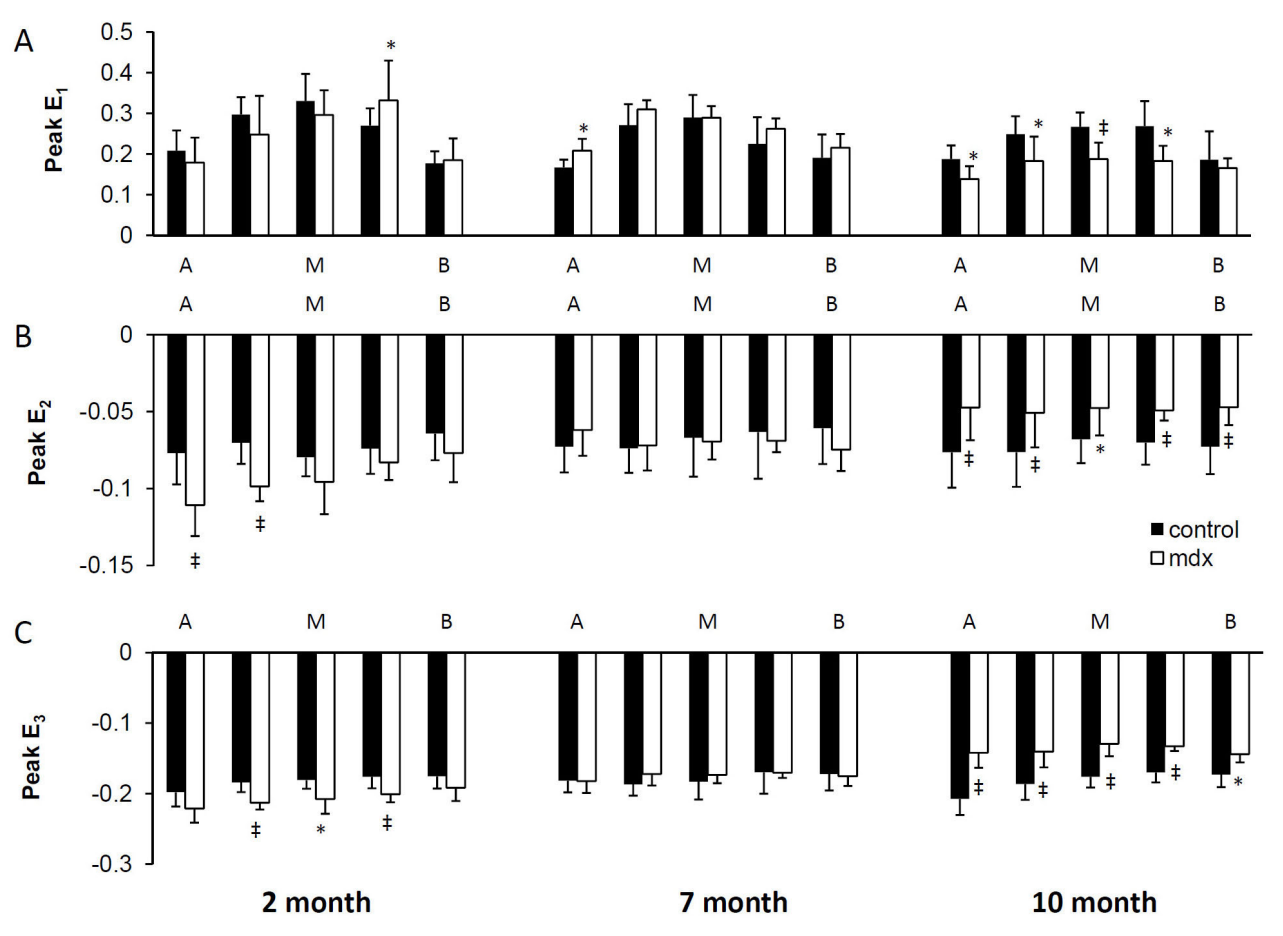
**Peak principal strains**. Peak E_1 _(A), E_2 _(B), and E_3 _(C) strains in 5 short-axis levels from apex (A), to mid-ventricle (M), and to base (B). *P < 0.05, ^‡^P < 0.01 between mdx mice and age-matched controls, respectively.

**Figure 4:**
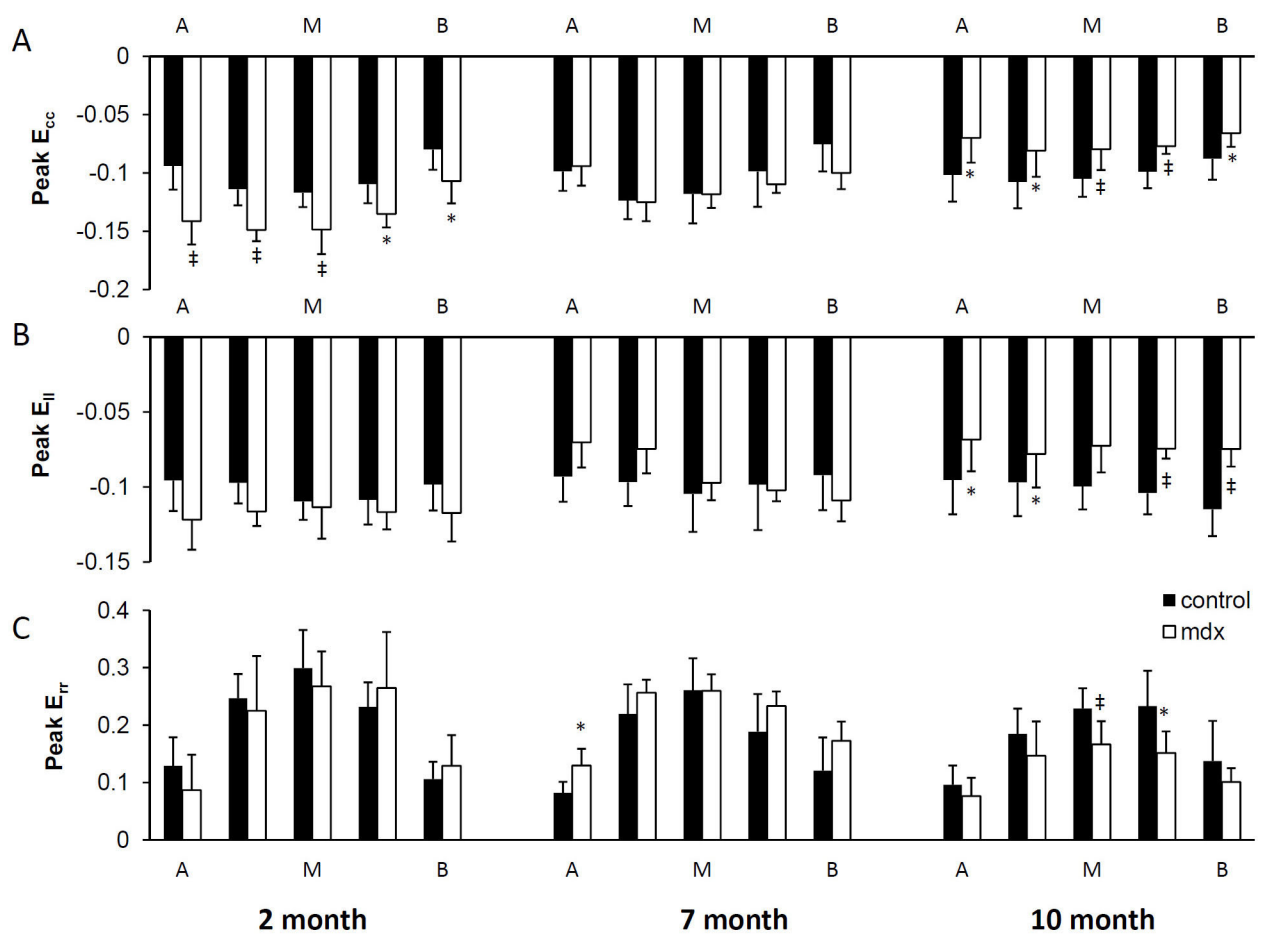
**Peak normal strains**. Peak circumferential (A), longitudinal (B), and radial (C) strains in 5 short-axis levels from apex (A), to mid-ventricle (M), and to base (B). *P < 0.05, ^‡^P < 0.01 between mdx mice and age-matched controls, respectively.

**Figure 5:**
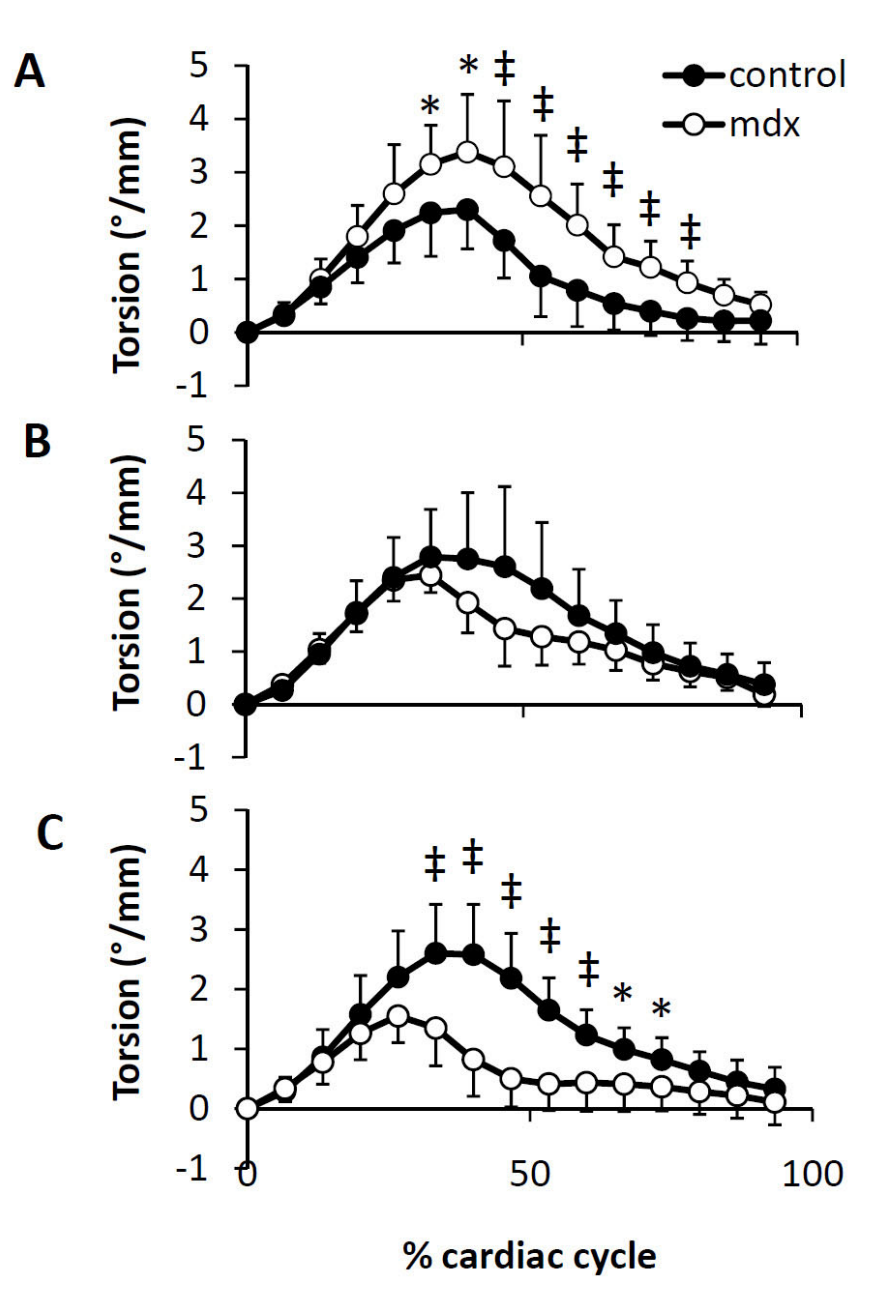
**Time course of ventricular torsion**. A. Increased ventricular torsion in 2-month mdx mice; B. Similar ventricular torsion between mdx and control mice at 7 month; C. Decreased ventricular torsion in 10-month mdx mice. *P < 0.05, ^‡^P < 0.01 between mdx mice and age-matched controls, respectively.

Peak principal strain, normal strain, and ventricular torsion are shown in Figs. 3, 4 and 5 respectively. 2-month mdx mice had significantly increased regional wall strain and torsion. Compared with the control mice, mdx mice had a 12-44% increase in E_2 _(P < 0.05 in two apical slices), a 9-16% increase in E_3 _(P < 0.05 in three midventricular slices), and a 27-50% increase in E_cc _(P < 0.05 for all 5 slices). Increased E_1 _was also observed in the slice between the mid-ventricle and the base in mdx mice (P < 0.05). Ventricular torsion was also significantly increased in mdx mice. The maximal torsion increased from 2.55 ± 0.41°/mm in control mice to 3.40 ± 0.83°/mm in mdx mice (P < 0.05).

### 7-Month Mdx Mice

7-month mdx mice had similar regional strain and time course of ventricular torsion as the controls. The maximal torsion of mdx mice was also similar to the controls (2.55 ± 0.25 °/mm v.s. 3.06 ± 0.97°/mm, P = 0.3).

### 10-Month Mdx Mice

10-month mdx mice had significantly decreased regional myocardial wall strains (E_1_, E_2_, E_3_, E_cc_, E_rr _and E_ll_) by 11~38% throughout the whole left ventricle, with the exception of E_1 _at base, E_ll _at midventricle, and E_rr _at both apex and base. The time course of ventricular torsion was significantly altered in mdx mice (Fig. 5C). The maximal torsion decreased from 2.65 ± 0.86°/mm in the control mice to 1.66 ± 0.40°/mm in mdx mice (P < 0.05).

### Histological Analysis and Principal Strains in Segmented Regions

**Figure 6:**
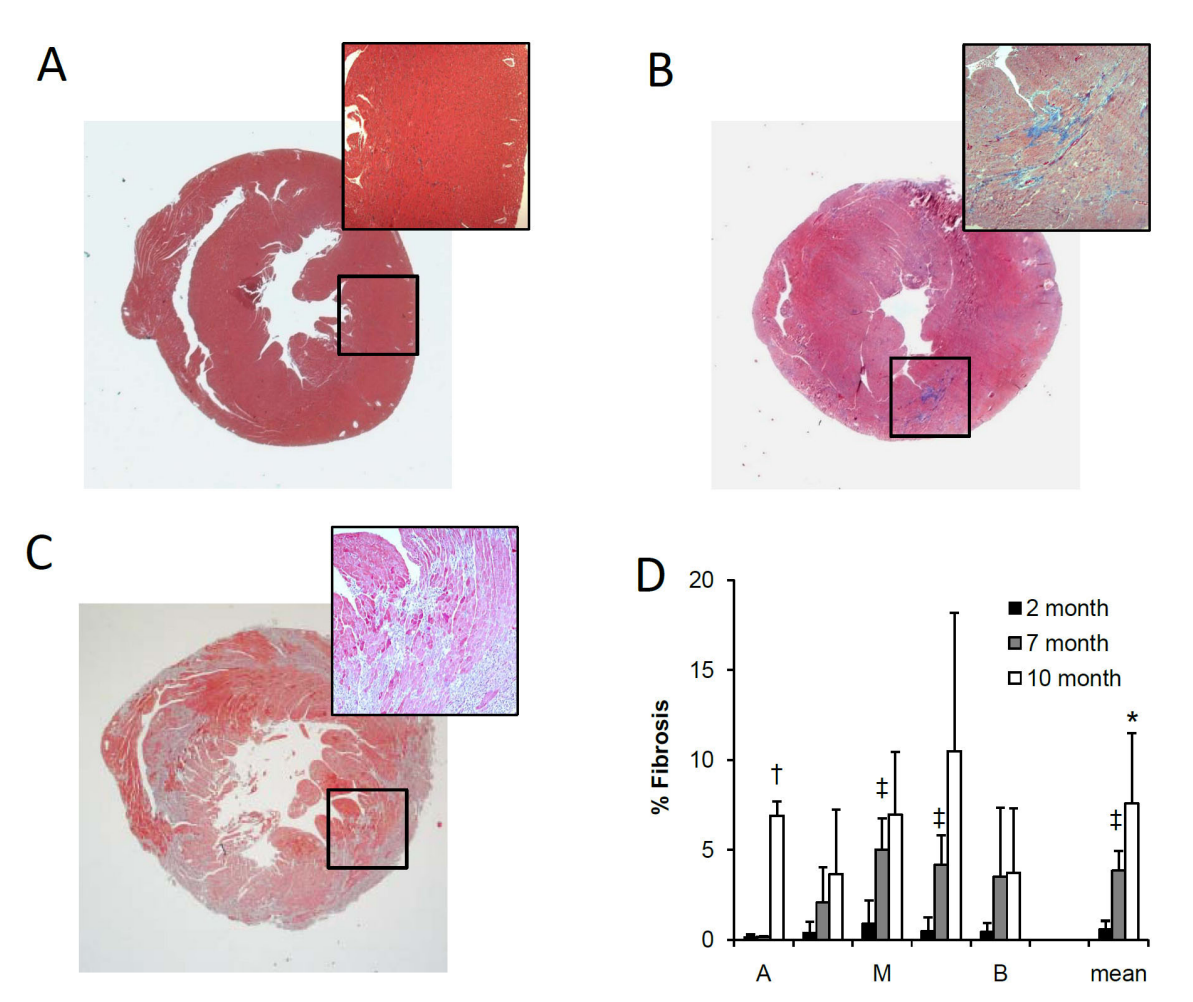
**Histological assessment of mdx mouse hearts**. Representative images of Masson's trichrome staining of mdx mouse hearts at 2 (A), 7 (B), and 10 (C) months of age. D. Quantification of fibrotic tissue at five short-axis levels from apex (A) to midventricle (M), and to base (B). ^‡^P < 0.01 between 2- and 7-month mdx mice. *P < 0.05, ^†^P < 0.01 between 7- and 10-month mdx mice, respectively.

Representative histological images are shown in Fig. 6. 2-month mdx hearts did not show obvious cardiac lesions (0.6 ± 0.5%). 7-month mdx hearts had mild interstitial fibrotic lesions (3.8 ± 1.1%). 10-month mdx hearts had a wide range of tissue fibrosis from interstitial to transmural fibrotic patches. The fibrotic tissue averaged 7.6 ± 3.9% in 10-month mdx mice. The fibrotic tissue displayed a random and scattered pattern. Myocardial fibrosis was observed in the septum, anterior, posterior and lateral segments of the left ventricle, as well as in the right ventricle.

**Figure 7:**
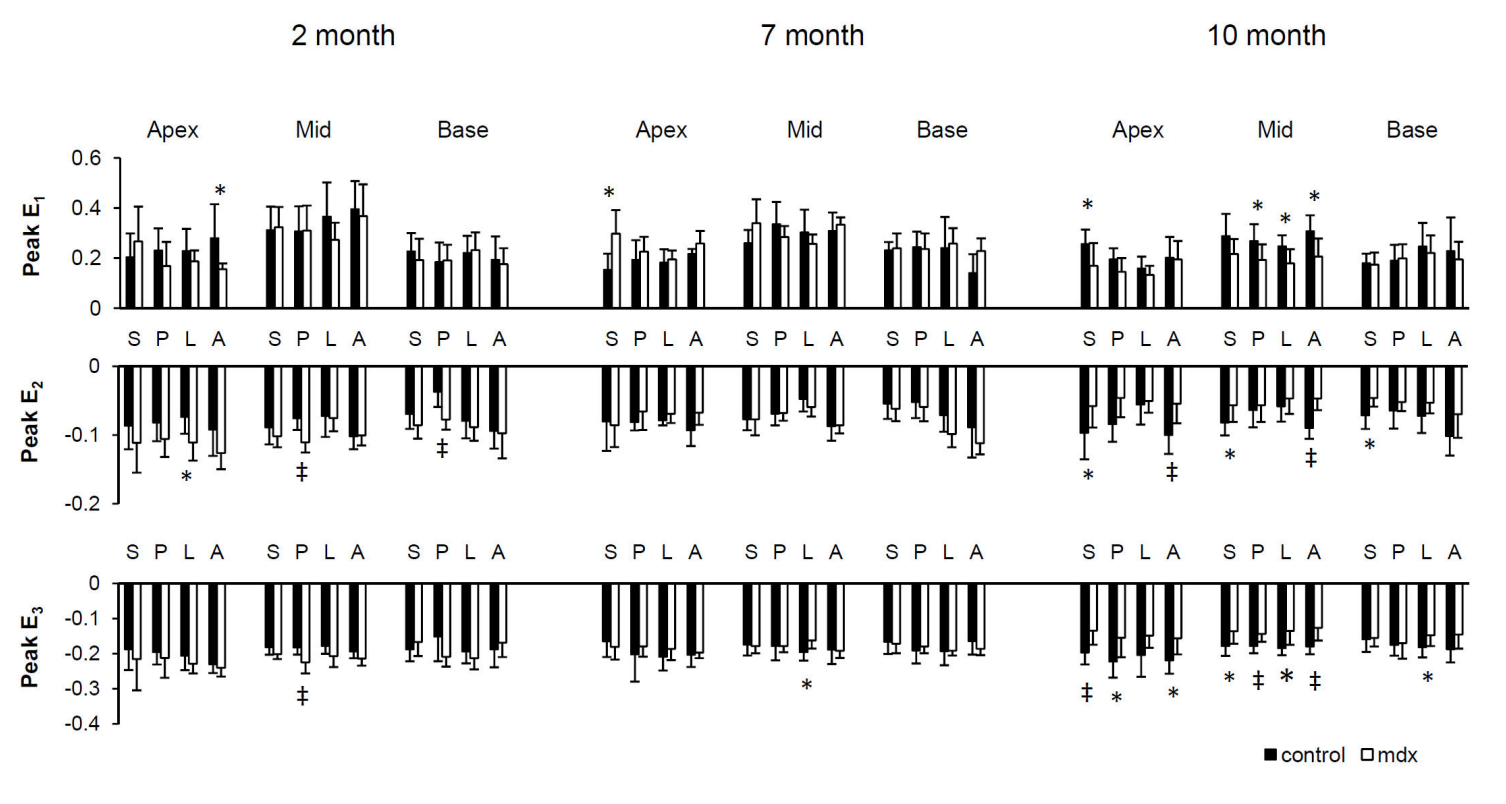
**Principal strains in segmented regions**. S, septum; P, posterior; L, lateral; A, anterior. *P < 0.05, ^‡^P < 0.01 between mdx mice and age-matched controls, prespectively.

Principal strains at apex, mid-ventricle, and base are shown in Fig. 7. For 2-month mdx mice, anterior E_1 _strain was decreased and lateral E_2 _strain was increased in the apex, posterior E_2 _and E3 strains were increased in mid-ventricle, as well as posterior E_2 _strain in the base (P < 0.05). For 7-month mdx mice, septum E_1 _strain was increased at the apex, while lateral E_3 _strain was decreased in mid-ventricle. At 10 months of age, all the segments showed either a decrease or a trend towards decrease in three principal strains in mdx mice. Consistent with the scattered fibrosis, all four segments of the left ventricle had a decrease in wall strain in 10-month mdx mice.

## Discussion

In this study in mdx mice, we assessed the alterations in regional left ventricular function at different stages of disease development towards DCM. Despite similar global functional indexes, both young and adult mdx mice manifested significantly altered patterns of regional ventricular wall motion. Consistent with the development of fibrotic lesions, myocardial wall strain and torsion were significantly decreased in 10-month mdx mice. However, the lesion-free young mdx mice had a surprising increase in regional wall strain and torsion, while 7-month mdx mice had similar contractile patterns as the controls.

Cardiac function has been investigated in mdx mice using both CMR and echocardiography. Young mdx mice exhibited normal morphology and global cardiac function [10,19]. At 8 months of age, mdx mice started to manifest right ventricular dilatation and decreased peak LV filling rate [11]. However, ejection fraction and cardiac output in mdx mice remained normal until 42 weeks of age [10,11]. While the normal global function in young and adult mdx mice is consistent with these previous findings, our study is the first to observe an early change in regional myocardial wall strain and torsion in mdx mice. Our results suggest that changes in regional wall motion can occur at a very early age in mdx mice before any macroscopic and global functional changes have occurred.

We observed a biphasic change in myocardial wall strain and torsion in mdx mice, with an initial increase at young age followed by decrease at older ages. Since dystrophin disruption renders mdx mice more vulnerable to mechanical stress and workload induced damage [20], the early increase in myocardial contraction may lead to increased ventricular wall stress in mdx mice, thus exacerbating stress-induced damage and triggering the initial remodeling processes that eventually leads to lesion development and cardiac dysfunction. The accumulation of tissue fibrosis leads to progressive decline in regional ventricular function in older mdx mice. As a result, 7-month mdx mice had similar regional ventricular function compared with the controls, while 10-month mdx mice had significantly decreased wall strain and torsion.

Cellular changes in mdx mice have also been investigated. In particular, the impact of dystrophin deficiency on Ca^2+ ^homeostasis has been investigated in both young (2-3 months) and adult (9-12 months) mdx mice by Williams et al [21]. They observed significantly increased Ca^2+ ^transient in both young and adult mdx mice, which was associated with increased expression of ryanodine receptor, the protein responsible for calcium release during systole. As Ca^2+ ^is the central regulator of myocyte contractility, it is possible that our observed functional increase in young mdx mice is attributable to enhanced myocyte contraction due to increased Ca^2+ ^transients. However, further investigation is needed to establish the relationship of calcium transients, myocardial contractile function, and disease progression in mdx mice.

The effects of increased Ca^2+ ^transients on myocardial remodelling have been investigated in other animal models. Increased Ca^2+ ^transients and myocyte hypertrophy were observed in mice with β_2_-adrenoceptor overexpression [22]. Increase in Ca^2+ ^transients via systemic β-agonist administration has also been shown to cause myocyte hypertrophy and apoptosis, tissue fibrosis, and heart failure [23]. All these studies indicate a possible mechanism leading to myocardial dysfunction via chronic elevation of Ca^2+ ^transients. Interestingly, altered Ca^2+ ^homeostasis was also observed in the skeletal muscle of both mdx mice and DMD patients [24-26]. These observations led to several clinical trials that evaluated the effects of Ca^2+ ^antagonists on DMD patients [27], with the primary focus on the skeletal muscle. In general, no significant beneficial effects were observed on the skeletal muscle function in DMD patients, although reduced muscle degeneration was revealed in several animal studies [28-30]. Only one study reported a significant improvement in muscle strength in verapamil-treated DMD patients, but with a high incidence of cardiac side effects [31]. Since Ca^2+ ^can impact both inotropic and chronotropic state of the heart, the effects of Ca^2+ ^antagonists on the heart need to be carefully examined.

Several studies have investigated LV function in DMD patients in search for early signs of myocardial dysfunction. Using CMR, Ashford et al reported normal LV volume and ejection fraction in young DMD patients. However, CMR tagging revealed significantly reduced circumferential strain in these patients [6]. Similar findings were reported using ultrasound-based methods. Mertens et al investigated a group of young DMD patients with preserved global function by echocardiography [32]. They observed significantly decreased peak radial and longitudinal systolic strain and strain rate, as well as peak systolic and early diastolic myocardial velocities in the anterolateral and inferolateral LV walls. Similarly, Mori et al reported decreased peak systolic radial strain and early diastolic wall-thinning velocity in the posterior LV wall of young DMD patients with normal conventional echocardiographic findings [33,34]. These studies suggest that DMD patients manifest decreased regional myocardial wall motion at an early age, which differ from the biphasic change we observed in mdx mice. Compare to DMD patients, the expression of disease in mdx mice is relatively milder [35]. One possible explanation for this difference between mdx mice and DMD patients is that a dystrophin analogue, utrophin, compensates for the lack of dystrophin more effectively in mdx mice than in DMD patients. Utrophin is associated with the DGC, and is similar to dystrophin in primary sequence and predicted secondary structure [36]. It can compensate for dystrophin when expressed at high levels. The severity of disease in mice lacking both dystrophin and utrophin is similar to DMD [37,38]. Further, a recent gene array study has confirmed the up-regulation of utrophin in mdx mice [39].

While the observed biphasic change in mdx mice suggests that tissue tagging is more sensitive for detecting functional alterations than global clinical indexes, functional assessment alone may not be sufficient in delineating disease progression when opposing effects exist. The seemingly "normal" regional wall motion in 7-month mdx mice reflects the balance between increased myocyte contractility and the development of myocardial fibrosis. Because of these counteracting factors, it is important to evaluate other aspects of myocardial remodeling. In particular, late gadolinium enhanced CMR (LGE-CMR) has been shown to detect myocardial lesions in DMD patients [40-42]. The combination of LGE-CMR and tissue tagging will provide more comprehensive evaluation of disease progression in DMD patients.

Despite significant alterations in ventricular torsion and wall strain at both 2- and 10-months of age, mdx mice showed no statistical difference in global ventricular function compared to the age-matched controls. The relationship between regional myocardial wall motion and global ventricular function is complicated because of the intricate 3D architecture of myocardial fibers. While CMR tagging measures the regional deformation of the myocardium, it remains to be elucidated to what extent altered myocardial deformation can be reflected in global changes in ventricular volumes. In general, current findings support the notion that regional alterations precede the manifestation of global functional changes. However, a more quantitative relationship between regional wall deformation and global ventricular function remains to be defined.

**Figure 8:**
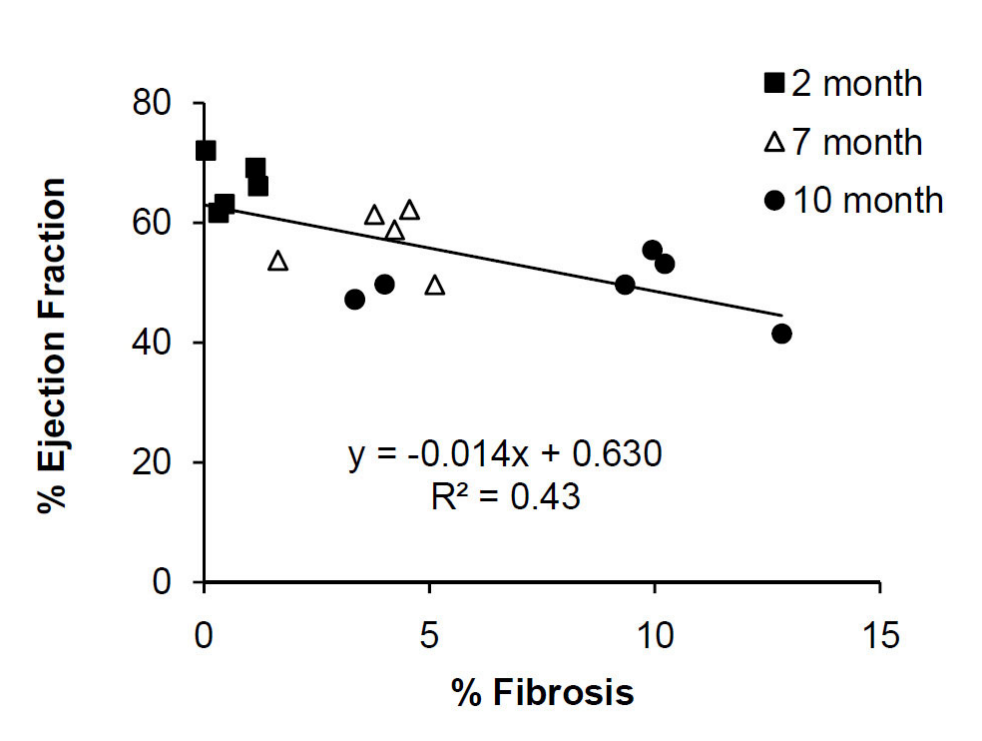
**Correlation between ejection fraction and myocardial fibrosis**.

Nevertheless, older mdx mice showed a trend towards decreased ejection fraction (Table 1). Correlation analysis revealed a weak, negative correlation between tissue fibrosis and ejection fraction (Fig. 8). A recent study of DMD patients also investigated the correlation between ejection fraction and myocardial lesion delineated by LGE [42]. Although a reduction in ejection fraction was reported in DMD patients with significant lesion development (21.9 ± 11.1%), no statistical significance was detected in age-adjusted correlation between ejection fraction and lesion size (P = 0.065). The mdx mice differ from DMD patients with delayed disease progression. With an average fibrosis of 7.6 ± 3.9% in 10-month mdx mice, the reduction in ejection fraction was insignificant. These data further suggest that analysis of regional myocardial wall motion can provide more sensitive measures of functional alterations in heart.

Previous LGE-CMR studies have identified the basal inferior and lateral walls as the initial sites of myocardial fibrosis [41,42]. A recent echocardiographic study also observed decreased radial strain in the posterior wall with relative sparing of ventricular septum [34]. However, such preferential changes were not observed by CMR tagging [6]. These discrepancies and the complicated relationship between myocardial fibre structure and regional function warrant further investigation. In the current study, we observed atypical patterns of lesion distribution and functional alterations in mdx mice. It remains to be investigated which pathogenic processes are responsible for the observed difference between mdx mice and DMD patients.

One limitation of the current study is that different animals were characterized in different age groups because of the requirement for histological analysis, which may potentially increase the variability of strain measurements. Heart rate variation during long image acquisition can be a source of error for the 3D reconstruction of the taglines. In the current study, this error was minimized by maintaining a constant heart rate with 4.1 ± 4.7% fluctuation. The TR was also adjusted according to the R-R interval such that each of the 15 frames was acquired at the same stage during a cardiac cycle. These measures can effectively minimize the errors due to heart rate fluctuation. The strain and torsion values in the control mice were similar to those reported in the literature [16,43].

Taglines were tracked semi-automatically with HARP-based method and homogeneous strain analysis was used for strain quantification in the current study. While the filtering of harmonic peaks in the k-space reduces the resolution of reconstructed HARP images, the accuracy of tagline tracking is not affected by k-space sampling/undersampling. The accuracy of homogeneous strain analysis was investigated previously using a deformable silicone gel phantom [44]. It has been shown that the error of noise-free strain estimation was within 10% of the model-calculated values, with no bias. With only one adjacent slice, strain quantification at apex showed a larger variation than at mid-ventricle as fewer tetrahedrons were generated. However, average strain values for each experimental group showed comparable standard deviations at apex, base, and mid-ventricle, suggesting that differences among animals were the dominant source of data variation.

## Conclusion

Despite normal global LV function, mdx mice had a biphasic change in myocardial wall strain and torsion, with an initial increase at young age followed by progressive decrease at older ages. These results suggest that regional LV function by CMR tagging is sensitive to early signs of myocardial dysfunction than global functional indexes. Therefore, CMR tagging can be utilized to detect early cardiac involvement, as well as to potentially monitor and quantify the effects of novel therapeutic interventions in DMD patients.

## Competing interests

The authors declare that they have no competing interests.

## Authors' contributions

WLi participated in study design, image and histological analysis, and prepared the manuscript. WLiu participated in study design, image acquisition and analysis. JZ participated in developing the 3D tagging analysis method. XY conceived and supervised the study and participated in the writing of this manuscript. All authors read and approved the final manuscript.
